# Pectin Dependent Cell Adhesion Restored by a Mutant Microtubule Organizing Membrane Protein

**DOI:** 10.3390/plants10040690

**Published:** 2021-04-02

**Authors:** Bruce D. Kohorn, Jacob Dexter-Meldrum, Frances D. H. Zorensky, Salem Chabout, Gregory Mouille, Susan Kohorn

**Affiliations:** 1 Department of Biology, Bowdoin College, Brunswick, ME 04011, USA; jldexter@bowdoin.edu (J.D.-M.); fdzorens@bowdoin.edu (F.D.H.Z.); skohorn@bowdoin.edu (S.K.); 2 IJPB, INRAE, AgroParisTech, Université Paris-Saclay, RD10, 78026 Versailles, CEDEX, France; Salem.chabout@inrae.fr (S.C.); gregory.mouille@inrae.fr (G.M.)

**Keywords:** cell wall, pectin, cell adhesion

## Abstract

The cellulose- and pectin-rich plant cell wall defines cell structure, mediates defense against pathogens, and facilitates plant cell adhesion. An adhesion mutant screen of Arabidopsis hypocotyls identified a new allele of *QUASIMODO2* (*QUA2*), a gene required for pectin accumulation and whose mutants have reduced pectin content and adhesion defects. A suppressor of *qua2* was also isolated and describes a null allele of *SABRE* (*SAB)*, which encodes a previously described plasma membrane protein required for longitudinal cellular expansion that organizes the tubulin cytoskeleton. *sab* mutants have increased pectin content, increased levels of expression of pectin methylesterases and extensins, and reduced cell surface area relative to *qua2* and Wild Type, contributing to a restoration of cell adhesion.

## 1. Introduction

The extracellular matrix (ECM) or cell wall of vascular plants is an interlaced array of cellulose, hemicellulose, pectin, and a variety of proteins that maintains cell structure, is a first barrier to pathogens, and provides a platform for cell adhesion [1,2,3]. Pectin is laid down in Golgi-derived vesicles between two dividing cells and after the elaboration of a complex cellulose and protein mixture proximal to the cell membrane, the pectin remains enriched in the middle lamella, an area between the two cells [4,5,6]. This pectin layer likely mediates cell adhesion, and its modification through de-esterification by esterases and their inhibitors [7], elaboration of side chains [8], or through cleavage, can alter the adhesive properties in a variety of organs [9].

Pectin is synthesized in the Golgi in an esterified form, and then selectively de-esterified in specific locations in the extracellular matrix by a large family of pectin methylesterase (PMEs) that are highly regulated transcriptionally [10]. Pectin methylesterase inhibitors (PMEIs), a family of 75 isoforms in Arabidopsis, are also tightly regulated and may interact in a pH dependent manner with specific PMEs to create a complex pattern of inhibition [11,12].

Polygalacturonases (PGs) hydrolyze pectin, loosen the cell wall, and allow for turgor-driven cellular growth, expansion, and cell separation [9]. PG activity requires pectin de-esterification and because PMEs are needed for calcium crosslinking, while also being required for PG activity, it is not surprising that PME activity increases cellular adhesion in some tissues, while decreasing it in others. For example, a high level of pectin methylesterification is correlated with an increase in cellular adhesion and decreased cell separation in tetraspores and root border cells respectively [13], while methylesterified pectin is correlated with a reduction in cellular adhesion in the mesophyll and pericarp [2,14,15]. These two opposing examples may reflect a different balance of PG and PME activity that change the relative amounts of degradation versus crosslinking and hence adhesion.

Mutations in putative Golgi-localized glycosyl and methyl transferases have supported the role of homogalacturonan (HG) pectin in cellular adhesion. The *QUA1* and *QUA2* genes encode a Golgi-localized glycosyl and methyl transferase, respectively. *qua1* and *qua2* mutants of Arabidopsis have a 50% decrease in HG pectin and show weakened cellular adhesion and cell detachment in the hypocotyl [16,17,18,19,20,21]. While the *qua* mutants show the importance of pectin abundance in cellular adhesion, other Arabidopsis mutants indicate that adhesion is not just dependent upon pectin abundance. A loss of function mutation in a putative Golgi-localized O-fucosyl transferase FRIABLE1 (FRB1) decreases adhesion without decreasing HG pectin abundance [20]. Instead *frb1* changes the amount of galactose- and arabinose-containing oligosaccharides in the Golgi, and alters pectin methylesterification, extensin, and xyloglucan microstructure. While it is hypothesized FRB1 fucosylates a protein in the Golgi [20], the precise target of FRB1 is unknown. It is likely that the *qua* and *frb1* mutants affect adhesion through the same pathway because the *qua2/frb1* double mutant does not show an additive phenotype [20]. While *frb1* shows complex changes to its cell wall, the mutant’s change in methylesterification may be directly responsible for its adhesion phenotype.

While mutations in *QUA1* [16], *QUA2* [19], and *FRB1* [20] lead to either a reduction in pectin levels (*qua1,2*) or a change in their esterification and modification (*frb1*) and a subsequent loss in cell adhesion, it is also likely that pectin crosslinking via extensins can affect the organization and adhesion of the cell wall [21,22]. Null alleles of *EXT3* are lethal and lead to general cell wall disorganization, and *EXT3* is upregulated in *qua2* mutants [21]. Studies using antiserum to a variety of extensin epitopes show a wide and varied distribution between tissue and species [20,23,24], and the relationship between specific genes and these epitopes is not well established. The identification of a putative fucosyl transferase ESMERALDA1 (ESMD1), whose mutant suppresses an adhesion defect in *qua2* and *frb1* without restoring pectin levels, points to the existence of an uncharacterized signaling mechanism that controls cell adhesion [25]. Thus, while pectin and its modification contribute to cellular adhesion, there are numerous other factors that are likely involved. To further our understanding of plant cell adhesion, a population of ethyl methanesulfonate (EMS) mutagenized Arabidopsis were screened for hypocotyl adhesion defects. This work describes the isolation of a new allele of *qua2*, and of a suppressor of *qua2* that is a new allele of *SAB* that encodes a previously described membrane protein required for microtubule organization [26,27]. The results also further support the essential role of pectin and its modification in cellular adhesion.

## 2. Results and Discussion

**Figure 1:**
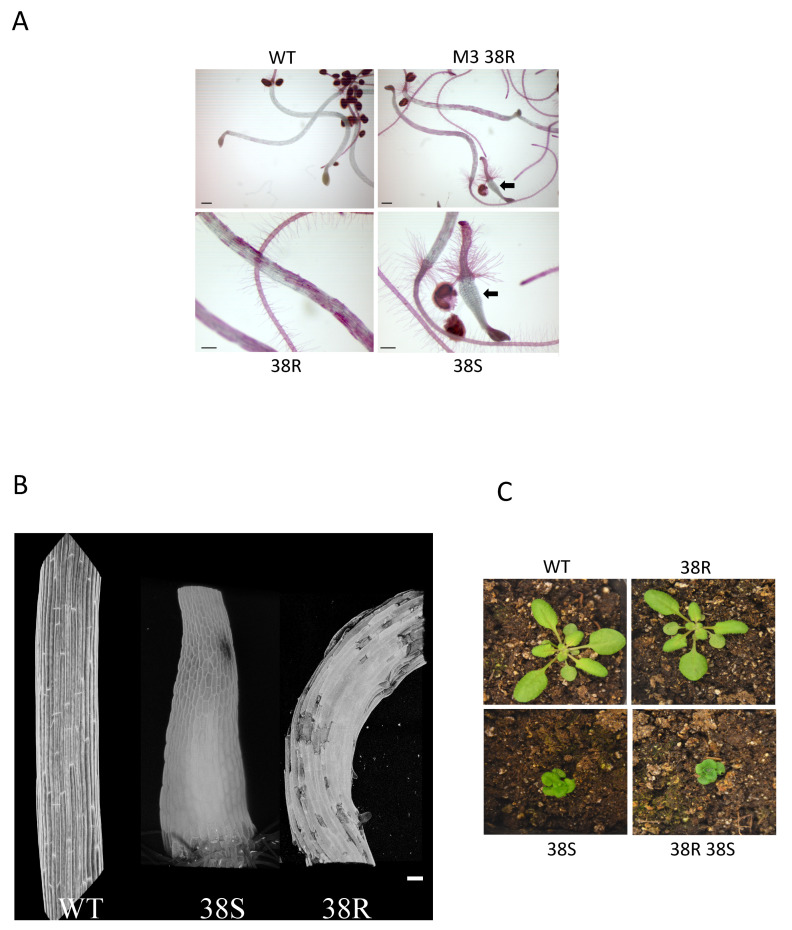
Cell adhesion mutants. (**A**) Dark grown seedlings of the indicated genotype were stained with ruthenium red. Arrow indicates the 38S isolate, lower panel is enlarged from the upper left. Bar indicates 1 mm upper, and 0.5 mm lower panel. (**B**) Confocal image of adhesion mutants. Propidium iodide was used as a general cell surface stain and detected by emission at 600–650 nm. Shown are four-day-old dark grown Wild Type (WT), 38S, and 38R hypocotyls. Only a portion of the hypocotyl is shown for WT and 38R. Bar indicates 100 mm. (**C**) Adhesion mutant suppressor is dwarf on soil.

Ruthenium red binds to de-esterified pectin, but while it cannot penetrate the cell wall of wild type Arabidopsis hypocotyls, it can penetrate defective cell walls as it does stain hypocotyls of adhesion mutants *qua2-1* and *frb1-2* [19,25] (Figure S1). Both mutant and wild type (WT) roots stain with ruthenium red. To identify new mutants in adhesion, dark grown seedlings of the M2 generation of ca. 5000 EMS mutagenized Arabidopsis seeds were screened for abnormal ruthenium red hypocotyl staining and visible cell detachment, and six mutants were identified (Figure S1). In one mutant, 38Red, (38R, Figure 1A), visible cell detachment, an irregular hypocotyl surface, and ruthenium red staining is shown in Figure 1A. To more closely view the cells of the hypocotyl, propidium iodide was used as a general hypocotyl cell surface stain, a three-dimensional image was created using confocal microscopy, and the results are shown in Figure 1B. Confocal microscopy of propidium iodide stained hypocotyls showed that cells are misplaced and curled (Figure 1B). In the M3 generation of a 38R self cross, only six out of seven seedlings had hypocotyls staining red and a new non-staining phenotype with a short, swollen root and hypocotyl appeared in the remaining seventh (Figure 1A, 38R arrow, and 38S). This new stumpy mutant called 38 short (38S) was expected to be homozygous for the 38R allele causing red staining, but also to carry an additional allele that suppressed red staining. In order to verify this, the 38R mutation was first characterized.

The 38R appeared similar to WT on soil (Figure 1C) and produced healthy siliques. The red staining M3 seedling was transferred to soil and then crossed to WT and F1 hypocotyls from this backcross (38R^+/−^), which are shown in Figure S2 and do not stain with ruthenium red, which indicated that the mutation responsible for the 38R adhesion phenotype is recessive.

**Figure 2:**
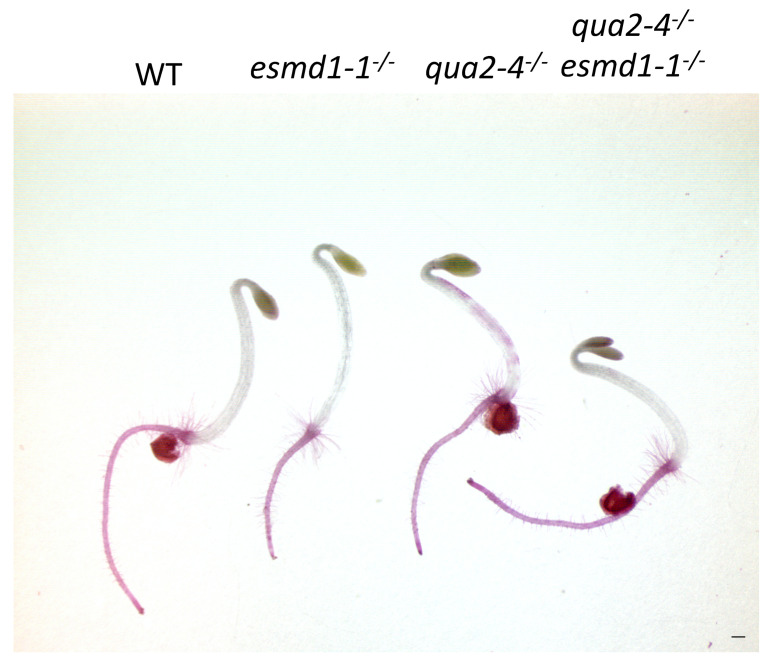
*esmd1* suppresses *qua2-4*. Dark grown seedlings of the indicated genotype were stained with ruthenium red. Bar indicates 5 mm. WT; Wild Type.

The 38R has a phenotype similar to but weaker than *qua2-1*, and polymerase chain reaction (PCR) -based sequencing shows that 38R is homozygous for a new *qua2-4* allele, causing a change of a conserved glycine to a glutamic acid at amino acid 580 in the amino transferase domain (Figure S3) [19]. All F1 progeny of a 38R^−/−^ crossed with *qua2-1* stain with ruthenium red, indicating that *qua2-1* fails to complement 38R (Figure S4A). PCR-based sequencing of these F1 show that the 38R and *qua2-1* alleles appear heterozygous as expected (Figure S4B). The *qua2-4*^−/−^ plant was then crossed with *esmd1-1*^−/−^ and F2 progeny homozygous for both loci were identified by PCR and sequencing of the two loci. Figure 2 shows that dark grown hypocotyls of *qua2-4*^−/−^ *esmd1-1*^−/−^ do not stain with ruthenium red, indicating that *esmd1-1*, that suppresses both *qua2-1* and *frb1-2* [25], also suppresses *qua2-4* (Figure 2).

The 38R M3 population also contained a seedling, 38S, that was stunted and did not stain with ruthenium red (Figure 1A, arrows). Because the 38S phenotype initially appeared in 8 out of the 58 plants observed and not in the expected one quarter of the population, and all M3 seeds germinated, the allele responsible for the 38S phenotype likely causes male or female gametophyte lethality. On soil, the 38S mutant exhibited short shoots, smaller irregularly shaped leaves, and did not produce functional siliques (Figure 1C). The *QUA2* gene from 38S leaves was PCR amplified and sequenced and M3 38S was homozygous for *qua2-4*, indicating 38S carries a suppressor of *qua2-4*.

Confocal microscopy of M3 38S propidium iodide stained hypocotyls shows that both the hypocotyls and the cells of the hypocotyl appear smaller than WT, and bulge outward relative to WT (Figure 1B). This is consistent with a loss of longitudinal expansion [28], but this was not quantified further since genetic analysis revealed that 38S is a well characterized mutant (see below).

**Figure 3:**
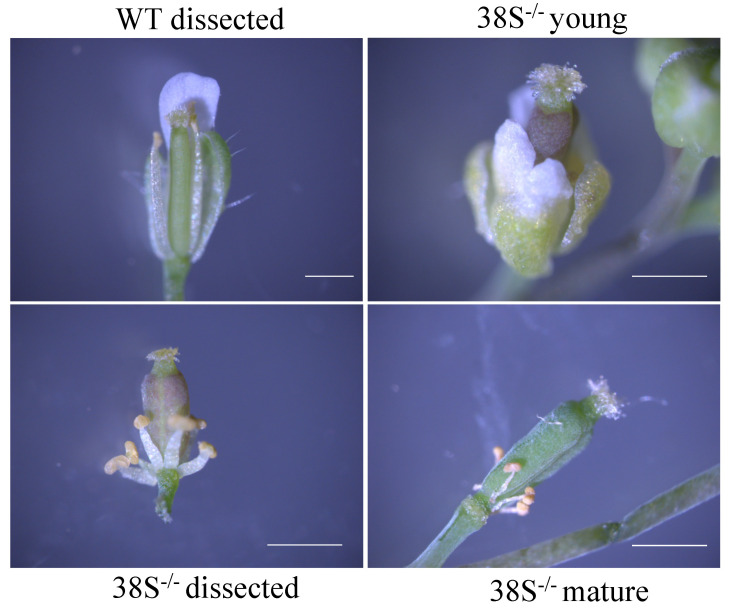
The stamen filaments of 38S have reduced growth. WT (2.5× mag) and 38S (4× mag) were dissected to reveal organs. The 38S young (4× mag) with intact flowers and 38S mature (4× mag) were not dissected. WT; Wild Type. Bar indicates 0.5 mm.

M3 38S did not produce normal siliques, and to evaluate this further the flowers were visualized by microscopy and partial dissection, and the results are shown in Figure 3. The 38S petals and sepals are malformed (Figure 3, 38S^−/−^ young), and as 38S flowers matured, the stamens remained short and withered (Figure 3, 38S^−/−^ mature). Thus, the lack of normal siliques is likely due to a lack of stamen elongation and the inability of pollen to reach the stigma (Figure 3). The 38S pollen is fertile because malformed stamens of 38S can be used to pollinate wild type (backcross) to produce heterozygous 38S F1. However, even when fertilized with WT pollen, 38S flowers did not produce siliques, indicating that the female infertility of 38S likely results from abnormalities in gynoecium maturation and/or female gamete production.

To determine if the 38S mutation was recessive, the hypocotyls of the F1 progeny of the 38SxWT (backcross) were examined by microscopy and ruthenium red staining and the results are shown in Figure S2. All F1 appeared wild type and hence the allele is recessive (Figure S2, 38S^+/−^). As expected, one seventh of the F2 generation of this backcross appeared stumpy when grown on agar in the light or in the dark in liquid. To determine if the 38S phenotype required the presence of the *qua2-4* allele, the *QUA2* gene was sequenced from eight stumpy seedlings (38S^−/−^) that appeared in the F2 generation of a 38S^−/+^ x WT. Of these eight stumpy seedlings, four were homozygous for *qua2-4*, three were heterozygous *qua2-4*, and one was homozygous WT. This result confirmed that the 38S stumpy phenotype does not require the *qua2-4* allele (Figure 1C).

To identify the mutation responsible for the 38S phenotype, 38S was backcrossed to WT, the F1 was then self crossed, and the F2 generation was planted on soil or in liquid. As expected, 101 of the 700 progeny showed a stunted (38S homozygote) growth phenotype. Leaves from 101 stunted F2 mutants from soil were pooled, and separately 115 dark grown mutant hypocotyls from liquid were pooled and DNA was then extracted from each pool and sequenced. Because only stunted plants were sequenced, all were necessarily homozygous for the stunted allele, and therefore during sequencing the causative mutation would be expected to appear with 100% frequency. Most other background mutations would be expected to segregate and appear with less than 100% frequency [29].

The pooled genome sequence from 101 38SxWT F2 38S stunted soil grown plants was analyzed using artMAP [29], which calculates allele frequencies and maps these to the Arabidopsis genome. The analysis indicated that nine mutations appear with 100% frequency in this F2 pool (Table 1). In the DNA sample from the hypocotyl pool, all of the same mutations as in the leaf pool were detected, except a mutation in mitochondrial DNA was found and there were no mutants on chromosome 3 at 100% frequency. The only mutation that appeared with 100% frequency and was predicted to have an amino change within the coding region of a gene was the change from a cytosine to a thymine that appeared within the *SABRE* gene (At1g58250). The mutation lying in the *SABRE* (*SAB*) gene introduces a premature stop codon at amino acid 455 of the 2655 amino acid long multi-transmembrane protein that has been previously shown to organize tubulin and is responsible for longitudinal cellular expansion [26,27]. Like 38S, hypocotyls of the previously characterized *sab* mutants do not elongate, and *sab* mutants exhibit a dwarfed phenotype highly similar to 38S [26]. A *sab1-5^−/+^* having a TDNA insertion that causes a null allele was crossed with a 38S^−/+^ [26] (note that neither homozygotes can be fertilized), and if the 38S and *sab* mutations were in different genes then all F1 should appear WT. Since 6 of 22 F1 seedlings (Figure S5, arrows) showed the stunted phenotype while the remainder appear similar to WT, *sab1-5* does not complement 38S and hence the mutations are in the same gene.

**Figure 4:**
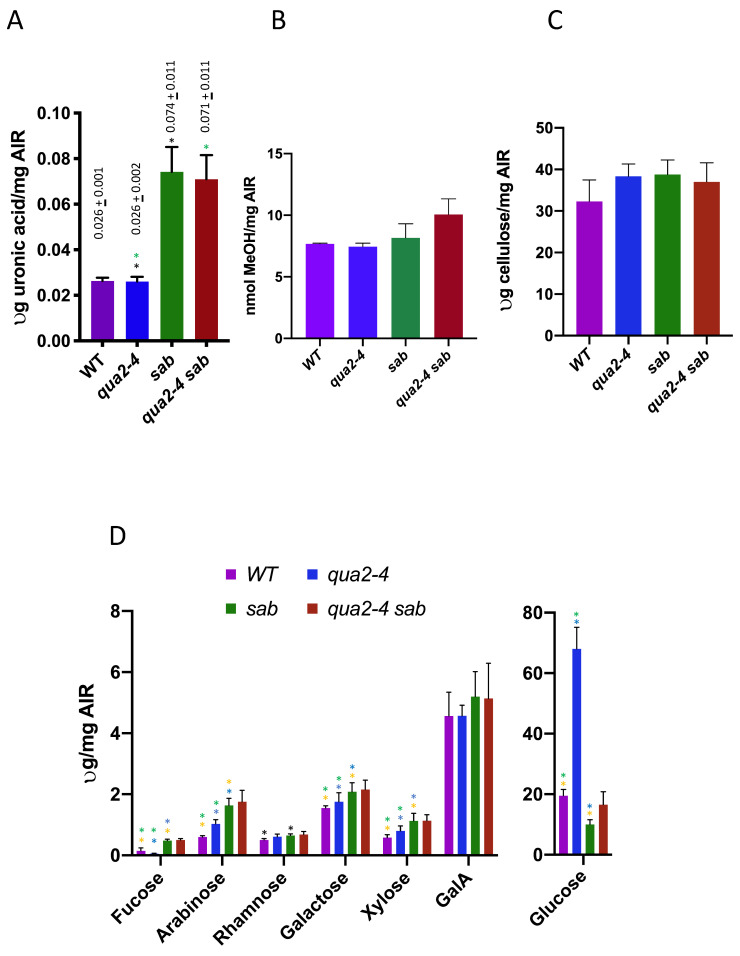
(**A**) *sab^−/−^* has increased pectin, and numerous other polysaccharide increases. Total uronic acid levels in µg/mg of alcohol insoluble residue (AIR) in the indicated genotype, measured from three biological replicates. Mean and standard deviation are shown above each bar. Similar colored asterisks indicate a significant difference (*t* test) between two genotypes within one type of measurement. Degree of methylesterification measured by the release of methanol from AIR is shown in (**B**). Cellulose (**C**) and the indicated sugars (**D**) were measured per mg of AIR using HPAEC-PAD chromatography. GalA; Galacturonic Acid.

*qua2-1* causes a 50% reduction in pectin content but no other cell wall alterations and this deficiency is thought to explain the loss of cell adhesion [19], and since *qua2-4* is a weaker allele, it was also expected to only reduce pectin levels, and have no other cell wall changes. The restoration in cell adhesion by *sab1* could be due to an increased level of pectin in *sab1* mutants relative to WT, or through a reduced cell surface area that requires less pectin for sufficient adhesion. To determine how *sab1* might suppress *qua2*, the pectin content was assayed by measuring ammonium oxalate extracted uronic acid in leaves of *qua2-4^−/−^, sab^−/−^qua2-4^−/−^*, and *sab^−/−^* (insufficient material can be collected from stunted hypocotyls). An ammonium oxalate enrichment is necessary to detect reduced pectin in *qua2-1*, as pectin changes in mutants such as *qua2* are detected only after ammonium oxalate enrichment but not in cell wall alcohol insoluble residue (AIR) fractions [19]. The results are shown in Figure 4, and both *sab^−/−^* and *sab^−/−^ qua2-4^−/−^* show several fold higher levels of galacturonic acid than do WT and *qua2-4^−/−^* (*t* test, *p* < 0.01, green and black asterisks). Thus, *sab1* over-compensates for pectin levels and thereby rescues a possible pectin deficiency caused by *qua2-4*. However, no difference was detected in pectin levels between WT and *qua2-4^−/−^* (*t* test, *p* > 0.01), and while this was not expected since *qua2-4^−/−^* does affect adhesion (albeit less than *qua2-1*) and is an allele of a gene required for pectin abundance, this method may be insufficiently sensitive to detect a small reduction especially in leaves. In addition, the *qua2-1* allele shows no reduction in leaves and previous differences with WT were only detected in dark grown hypocotyls [16,19,25]. The level of pectin methylesterification was also determined by measuring the amount of methanol released from NaOH treated AIR preparations, and there are no differences between WT and the three mutant genotypes (*t* test *p* > 0.05, Figure 4B). Since *sab^−/−^* and *qua2-4^−/−^ sab^−/−^* have elevated levels of pectin, this implies that the level of pectin esterification is reduced in *sab^−/−^* and *qua2-4^−/−^ sab^−/−^.*

Total cell wall composition was also determined for *sab^−/−^* and *sab^−/−^ /qua2-4^−/−^* mutants by trifluoroacetic acid treatment of AIR and quantitation of the released monosaccharide using High-Performance Anion-Exchange Chromatography with Pulsed Amperometric detection (HPAEC-PAD), and the results are shown in Figure 4C,D. No differences are detected in cellulose (*t* test *p* > 0.05, Figure 4D). In *sab^−/−^* relative to WT, fucose, arabinose, galactose, glucose, and xylose are all increased (*t* test, *p* < 0.05; orange asterisk). The fucose amount is lower and arabinose, galactose, xylose, and glucose are increased in *qua2-4^−/−^* relative to WT (*t* test, *p* < 0.05; green asterisk), and relative to *qua2-4^−/^, sab^−/−^* is only higher in fucose, galactose, arabinose, and glucose (*t* test, *p* < 0.05; blue asterisk). The dramatic increase in glucose in *qua2-4^−/−^* likely reflects the accumulation of starch as previously observed in leaves [16]. There were no differences detected between *sab^−/−^* and *sab^−/−^ /qua2-4^−/−^* for any cell wall components. Thus, *sab^−/−^* causes a significant change in multiple cell wall polysaccharides that may have a role in rescuing the *qua2-4^−/−^* phenotype.

**Figure 5:**
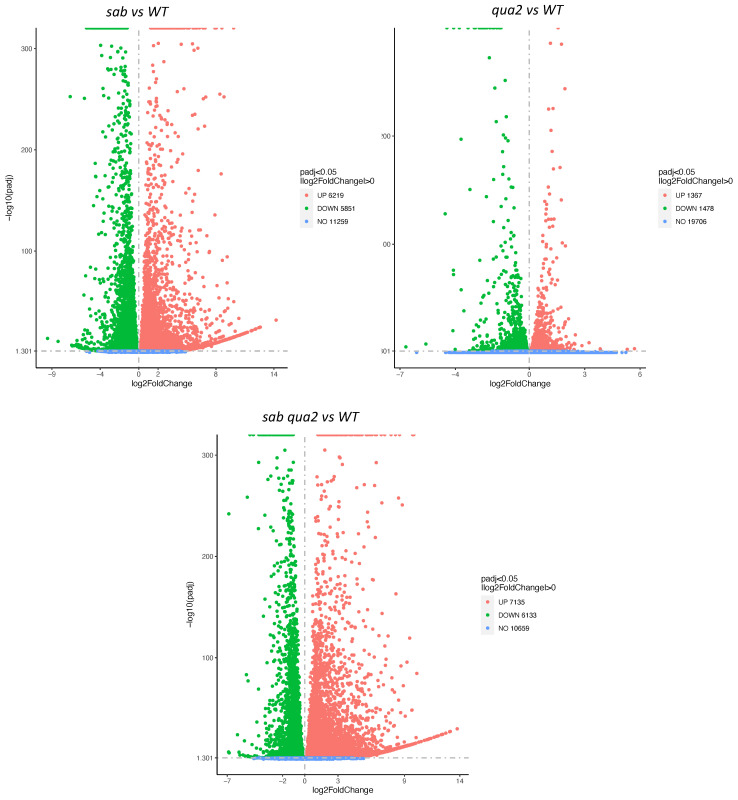
Volcano plot of differentially expressed genes in the indicated genotype as determined by RNA Seq. See Figures S6 and S7 for data.

**Figure 6:**
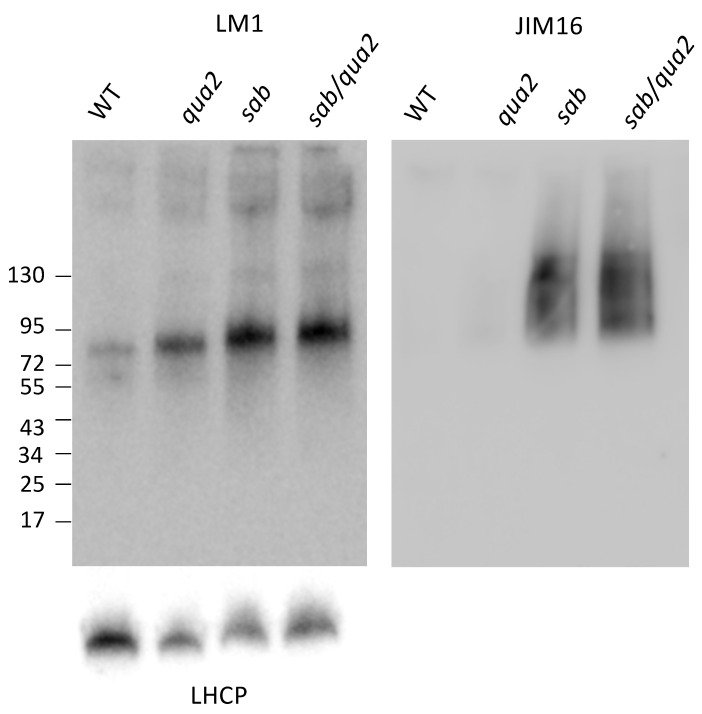
Extensin and arabinogalactan protein (AGP) epitopes are increased in *sab* mutants. Shown is a Western blot using anti extensin LM1 and anti AGP JIM16 antisera against equal amounts of total leaf protein from the indicated genotype. Bottom panel is a Western blot of similar samples probed for anti-light harvesting protein (LHCP) as a loading control. Numbers on left indicate MW x10^−3^.

To determine if the cell wall changes in the *sab* mutants might be reflected in gene expression changes, and therefore predict biosynthetic changes, a comparative RNA Seq analysis was performed on mRNA from soil-grown leaves. Significant differences were detected in gene expression between *sab^−/−^* and WT, and as expected *sab^−/−^* and *sab^−/−^qua2-4^−/−^* expression patterns are more similar to each other (Figure 5, Figure S7), and Gene Ontology (GO) analysis indicates that most of these changes are due to ribosome biosynthesis and metabolism. Surprisingly, the regulation of genes encoding pectin biosynthesis and degradation, and proteins involved in monitoring cell wall integrity appear not to be dramatically altered in any of the mutants (Figures S6 and S7 Cell Wall related sheet). While 5 glycosyl hydrolases are slightly upregulated, these are not known to affect pectin. The Wall Associated Kinases (WAKs) that are likely pectin receptors, appear slightly upregulated, but these are often increased by numerous stresses and it is not yet known what an increased mRNA level indicates [1]. However, there are two cell wall-related gene families that do show dramatic changes. *sab^−/−^* and *sab^−/−^qua2-4^−/−^* do have a 5–10 fold (log 2, *p* adj < 0.05) increase in expression relative to WT of multiple pectin methylesterases (PME) and their inhibitors (PMEI) that regulate the charge induced crosslinking of pectin (Figures S6 and S7), presumably in part to compensate for increased pectin levels. However, further analysis of the complex interplay between the expression and interaction between PMEs and PMEIs is needed to understand how these particular changes might directly affect pectin [11,12]. In addition, two extensins, EXT3 and EXT4 mRNAs, are highly upregulated suggesting that the crosslinking of pectin to cellulose is increased. This also may well be to compensate for a reduced level of adhesion and is consistent with the increased arabinose content in the cell wall (Figure 4C). While EXT3 mRNA is slightly up regulated in *qua2* mutants [21], the *sab1* allele causes a dramatic 8-fold (log2) change. Western blots using LM1 antiserum [23,24] indicate that the extensin protein levels are also increased by 5-fold relative to WT (Figure 6), but not as significantly as the EXT3 and EXT4 mRNAs. The molecular weight of the extensin is slightly increased (Figure 6) in *sab^−/−^* and *sab^−/−^qua2-4^−/−^* relative to *qua2^−/−^* and WT suggesting that the carbohydrate chain length or number is increased, and this has been observed in other conditions [22,30]. Since extensins can become crosslinked in the wall, the absolute increase in extensin protein levels might not be detected by Western blots and this may explain why the protein levels do not increase as dramatically as do the mRNAs. Moreover, although the EXT mRNA levels are increased in the *sab* mutants and predict an increase in EXT protein, since the LM1 epitope includes protein, galactose, and arabinose [23,24], the increase in the Western signal may also indicate a change in the carbohydrate moiety. However, a direct link between the LM1 epitope and EXT3 and 4 is not demonstrated by these results, and the specific EXT affected has not been determined. In addition to the large increase of EXT3 and EXT4 mRNA, three members of the arabinogalactan protein (AGP) family, AGP2, AGP5, and AGP17 mRNA are induced 1–5-fold (Figures S6 and Figure S7, log2, *p* adj < 0,05). Western blotting with JIM16 antiserum that detects arabinogalactan proteins (AGPs) [24] also shows a dramatic increase in this epitope in *sab^−/−^* and *sab^−/−^ qua2-4^−/−^* mutants, relative to *qua2-4^−/−^* and WT (Figure 6). Since the antiserum is detecting both the arabinogalactan and protein [24], the increase seen on the Western blot likely reflects both the increase in protein predicted by the mRNA levels, and a change in modification consistent with the changes in arabinose, rhamnose, and fucose detected in the cell wall (Figure 4D) as these sugars are covalently bound to AGP. However, a direct link between the JIM16 epitope and specifically AGP2, AGP5, and AGP17 has not been demonstrated by these results, and the specific AGP affected has not been determined.

In summary, the screen for new mutants in adhesion identified a new allele of the putative Golgi-localized pectin methyltransferase QUA2, where a change from a glycine to a glutamic acid leads to a mild loss of adhesion. The screen also identified a premature stop codon within the *SAB* gene that suppresses the *qua2-4* adhesion phenotype. SAB is a widely expressed, hydrophobic membrane protein that has previously been shown to organize ROP2/4 and the microtubule binding protein CLASP leading to an alignment of microtubules to promote longitudinal cell elongation [27,31,32,33]. Since oryzalin induced reduction in growth and cortical microtubule organization does not rescue the adhesion defects of *qua2* [21], the reduced cell size and previously demonstrated microtubule reorganization in *sab* [26,27] would be by itself unlikely sufficient to explain the suppression of the *qua2* adhesion phenotype. Rather, the higher levels of pectin in *sab1^−/−^* and *sab1^−/−^qua2-4^−/−^* can explain the restoration of adhesion in *qua2*. The observation that *sab* mutants have reduced pectin methylesterification increases the potential for calcium-mediated crosslinking, which too might contribute to the rescue of adhesion. *sab1* appears not to alter pectin abundance by altering the transcription of pectin biosynthesis genes, suggesting that the pectin levels may be higher in *sab^−/−^* due to a reduced turnover of pectin. However, no significant increase in expression of genes encoding pectin-degrading enzymes was detected. The exploration of pectin transport may also reveal how SAB might control pectin accumulation. Mutations in kinesin Fragile Fiber 1 (FRA1) [34], and SPIKE1, which controls lateral microtubule clustering [35], likely affect the trafficking of pectin-containing Golgi [36] but these mutants do not show pectin changes or adhesion defects. Thus, SAB may have a specific effect on the accumulation of pectin in the cell wall.

Genes encoding proteins for pectin modification and crosslinking are however highly upregulated in *sab1^−/−^* and *sab1^−/−^qua2-4^−/−^*, including *EXT 3* and *EXT4*, and while it is not known if the proteins encoded by these particular genes are involved in the *sab* suppression, the EXT family of proteins are thought to mediate pectin crosslinking to other wall components such as cellulose [21,22,30]. Thus, while pectin levels are higher and esterification rate is lower in *sab1^−/−^* and *sab1^−/−^qua2-4^−/−^* and this can explain the restoration of adhesion and lack of ruthenium red staining, the elevated levels of extensin and AGPs might also be responsible as this may increase the level of crosslinking of wall components. The work here provides further evidence that both the amount, and the levels of pectin modification and crosslinking agents can have dramatic effects on cell adhesion in Arabidopsis, and are directly influenced by a protein that has been previously shown [26,27] to be involved in the organization of the cytoskeleton.

### 3.1. Plant Growth Conditions

*Arabidopsis thaliana* seeds were sterilized for 5 min in 95% ethanol and then 5 min in 10% bleach and rinsed twice with sterile dH_2_O. Seedlings were then grown on agar containing Murashige and Skoog (MS) media (Sigma Aldrich, Saint Louis MO, USA) pH 5, with 2% agarose and 1% sucrose or planted directly onto soil. Following plating, seeds were exposed to cold (4 °C) for 48 h, grown at 20 °C for 10–14 days in 8 h of dark, 16 h of light. Seeds planted directly on soil were exposed to cold (4 °C) for 48 h and grown with a cover on. For in-experiment comparisons, samples were grown at the same time in triplicate. Plants were imaged using a Nikon D3000 camera. Total leaf area of each plant was measured using ImageJ.

### 3.2. Mutant Identification

To identify *Arabidopsis thaliana* mutants with abnormal cellular adhesion, approximately 5000 M1 seeds were mutagenized with ethyl methanesulfonate (EMS) [37] and grown on soil (M2 generation seeds were then collected in 191 pools (each pool contained the progeny of approximately 20–30 plants). Then, 100 seeds from each pool were then grown on liquid media after sterilization and stained with ruthenium red dye as follows. Liquid media was removed and 3 mL of ruthenium red dye (Sigma Corp. Saint Loui MO, USA, 0.5 mg/mL in dH_2_O) was applied to seedlings for 2 min in a 10 mL microtiter growth plate. After 2 min, seedlings were washed twice with 5 mL of dH_2_O. Hypocotyl staining was then observed under a dissecting microscope, and mutants were isolated and plated on MS agarose for 5 days before being transferred to soil.

### 3.3. DNA Extraction and PCR

Three-week-old healthy green leaves from plants of interest were collected, frozen in liquid N_2_, and DNA was extracted as described [38]. The indicated genes were PCR amplified according to the manufacturer’s conditions using Titanium Taq DNA polymerase (Takara Bio, Mountain View CA USA) using primers shown in Figure S2C, and samples were sequenced by Retrogen Corp.

### 3.4. 38 S F2 Whole Genome Sequencing

The pooled 38S F2 DNA preparations were sequenced with Illumina genome sequencing technology performed by Novogene. Analysis of the 38S F2 allele frequencies was performed using the data files provided by Novogene, and the programs artMAP (used to identify the allele frequencies) [29], and IGV (used to visualize the genome sequence) [39].

### 3.5. Cell Wall Preparation

Leaves from three biological replicates were immersed in 96% ethanol and incubated at 70 °C for 30 min, homogenized using a ball homogenizer for 20 min, centrifuged for 15 min at 20,000× *g*, and the supernatant was removed and the pellet was re-suspended in 100% ethanol and centrifuged for 15 min at 20,000× *g*. The supernatant was then removed and the pellet was re-suspended in methanol/chloroform (2v/3v) and shaken overnight. Samples were then centrifuged for 15 min at 20,000× *g* and the supernatant was removed. The pellet was then re-suspended sequentially in 100%, 65%, 80%, and 100% ethanol. After each re-suspension, samples were centrifuged at 20,000× *g* for 15 min and the supernatant was removed and the pellet or alcohol insoluble residue (AIR) dried under vacuum. The AIR was saponified overnight in 0.05 M NaOH and the samples centrifuged at 4 °C, 10,000× *g* 10 min. Methylesterification of the pectin was quantified by measurement of the methanol content in the supernatant [25]. The pellet was then washed twice with 70% ethanol (to remove residual NaOH) and twice with acetone at room temperature and dried under vacuum. Ammonium oxalate-extracted uronic acid content of the AIR was determined according to [25,40]. Neutral monosaccharide composition analysis of the non-crystalline polysaccharide fraction was performed after hydrolysis of a portion of the AIR in 2.5 M trifluoroacetic acid for 1.5 h at 100 °C, and the released monosaccharide quantified using HPAEC-PAD chromatography [25]. Cellulose content was determined through the hydrolysis of the rinsed residue resistant to TFA hydrolysis (crystalline polysaccharide) as described in [25].

### 3.6. Confocal Microscopy

Four-day-old dark grown seedlings were stained for 10 min with 10 υg/mL propidium iodide, and then washed one time in dH_2_0. Hypocotyls were then visualized by confocal microscopy on a Leica SP8 microscope using a 10× objective, a 514 nm excitation laser, and an emission spectra of 620–40 nm. A Z stack was then created for the seedling using the Leica SP8 software.

### 3.7. RNA Seq

*RNA seq* and bioinformatics was performed by Novogene Corp. (Sacramento CA USA) on biological triplicate, 3-week-old leaf RNA samples isolated using a Qiagen RNA isolation kit (Germantown MD.) Analysis of specific transcripts was carried out by Novogene using NOVASYSTEM online software which is R based.

### 3.8. Western Blotting

Leaves were ground with a pestle in 10 mM Tris 7, 3% sodium deodecyl sulfate, 100 mM Dithiothreitol, 10% glycerol, with 2X bromophenol blue loading dye and centrifuged at 10,000× *g* for 5 min. Prior to loading, samples were heated at 95 °C for 10 min. Samples were then separated using a 10–20% SDS-polyacrylamide gel and transferred to a nitrocellulose membrane for 1500 mA h. Following transfer, the blot was blocked in 5% non-fat dried milk in Tris buffered saline (20 mM Tris pH 7.5, 150 mM NaCl, 0.1% Tween-20) and subsequently incubated with anti-light harvesting protein (LHCP), LM1, or JIM 16 antibody at 1:2500 for 2 h at room temperature, washed in Tris buffered saline 0.3% Tween (TBST) and then subsequently incubated with horseradish peroxidase (HRP) conjugate anti-rabbit or rat secondary antibody (1:2500) for 2 h at room temperature. Blots were detected using SuperSignal Chemiluminescent detection kit (Thermo Fisher, Waltham MA, USA) and visualized using G-box (Syngene, Frederick MD, USA). Image J was used to quantify bands on the Western blots.

## Figures and Tables

**Table 1:** Allele frequency in the 38SxWT F2 pool of dwarf 38S leaves as determined by genomic sequencing and calculated by artMAP [29]. Shown are the alleles that occur at 100% frequency (indicated by 1). Only one mutation, in *SAB*, causes a significant change.

*Chromosome*	*Position*	*Mutation*	*Frequency*	*Gene Name*	*Gene ID*	*Protein Change*
1	2843028	G→A	1	*BON3*	*AT1G08860*	Leu63→Leu
1	19775799	C→T	1	*AT1G53060*	*AT1G53060*	Not in gene
1	21090506	C→T	1	*CRT1*	*AT1G56340*	Lys367→Lys
1	21597603	C→T	1	*SAB*	*AT1G58250*	Trp455→STOP
1	23861918	G→A	1	*AT1G64290*	*AT1G64290*	Not in gene
2	2782049	C→T	1	*AT2G06870*	*AT2G06870*	Transposon
2	3070506	C→T	1	*AT2G07390*	*AT2G07390*	Transposon
2	3638476	C→T	1	*AT2G09187*	*AT2G09187*	Transposon
3	9895262	G→A	1	*AT3G26840*	*AT3G26840*	Leu523→Leu

## Data Availability

All data is available in the manuscript or the supplementary materials.

## Supplementary Materials

Supplementary materials can be found at https://www.mdpi.com/article/10.3390/plants10040690/s1. Figure S1. Ruthenium red-stained M2 dark grown hypocotyls from the indicated seed pool. Shown are red staining seedlings isolated from 192 M2 pools of seeds of 20–33 plants. WT, *frb1*, and *qua2* are shown as controls. This report describes the characterization of mutant 38R, and 14, 152, 163, 241, and 242 will be reported elsewhere. Bar indicates 0.5 mm. Figure S2. 38R and 38S are recessive alleles. Shown are ruthenium red-stained dark grown hypocotyls of the F1 of the indicated mutant crossed to WT. Bar indicates 1 mm. Figure S3. CLUSTAL format alignment of QUA2 amino acid sequence to its two closest *Arabidopsis thaliana* methyl transferase homologous [41]. An asterisk indicates positions which have a single, fully conserved residue, a colon indicates conservation between groups of strongly similar properties, and a period indicates conservation between groups of weakly similar properties [42]. The location of *qua2-4* (G to E) is highlighted in green and *qua2-1* (I to STOP) is highlighted in red. Swiss-Model predicted structure of aa 532–678, which lie within the methyl transferase domain of QUA2, is shown to the left of the sequence. Hydrophobic regions are highlighted in white, while hydrophilic regions are highlighted in blue [43]. *qua2-4* (green dot at amino acid 580) lies within a long hydrophobic region of QUA2 and is characterized by a change from a glycine (aa structure shown) to a glutamic acid. Figure S4. *qua2-1* does not complement 38R adhesion defect. (**A**) 4-day-old ruthenium red-stained dark grown hypocotyls from the indicated genotype. (**B**) The *QUA2* gene was PCR amplified and sequenced from DNA of an F1 *qua2-1^−/+^ qua2-4^−/+^* seedling, and shown are the heterozygous loci for each allele. Figure S5. 38S and *sab1-5* are alleles of the same gene. Shown are dark grown hypocotyls from F1 seeds of a 38S^−/+^ x sab1-5^−/+^ cross. The presence of 1/7th of the population of stunted progeny (arrows) indicates the lack of complementation. Bar indicates 1 mm. Figure S6. Excel file of RNA seq. List of differentially expressed genes related to the cell wall compared between *sab* and WT. Similar levels are seen in *sab/qua* vs. WT. In addition, the following genes were evaluated and found not to differ significantly between genotype; Cell Wall Integrity—*FER1, HERK1, HERK2, FEI1, FEI2, KIN13A, THE1, WAK1-5*. Biosynthesis; *CESA, GAE1-6, PMT, PG, PGIP, PGX1, MNA, ORT2, APG*. Figure S7. Additional Excel sheets 2–4 show differential expressed genes (DEG) between WT and *sab*, *qua2*, or *sab qua2*.

## References

[1] Kohorn B.D. (2016). Cell wall-associated kinases and pectin perception. J. Exp. Bot..

[2] Wolf S., Hématy K., Höfte H. (2012). Growth Control and Cell Wall Signaling in Plants. Annu. Rev. Plant Biol..

[3] Somerville C., Bauer S., Brininstool G., Facette M., Hamann T., Milne J. (2004). Toward a systems approach to understanding plant cell walls. Science..

[4] Cosgrove D.J. (1997). Assembly and enlargement of the primary cell wall in plants. Annu. Rev. Cell Dev. Biol..

[5] Caffall K.H., Mohnen D. (2009). The structure, function, and biosynthesis of plant cell wall pectic polysaccharides. Carbohydr. Res..

[6] Daher F.B., Braybrook S.A. (2015). How to let go: Pectin and plant cell adhesion. Front. Plant Sci..

[7] Harholt J., Suttangkakul A., Scheller H.V. (2010). Biosynthesis of Pectin. Plant Physiol..

[8] Mravec J., Kračun S.K., Rydahl M.G., Westereng B., Miart F., Clausen M.H., Fangel J.U., Daugaard M., Van Cutsem P., Licht H.H.D.F. (2014). Tracking developmentally regulated post-synthetic processing of homogalacturonan and chitin using reciprocal oligosaccharide probes. Development.

[9] Yang Y., Yu Y., Liang Y., Anderson C.T., Cao J. (2018). A Profusion of Molecular Scissors for Pectins: Classification, Expression, and Functions of Plant Polygalacturonases. Front. Plant Sci..

[10] Sénéchal F., Wattier C., Rustérucci C., Pelloux J. (2014). Homogalacturonan-modifying enzymes: Structure, expression, and roles in plants. J. Exp. Bot..

[11] Sénéchal F., L’Enfant M., Domon J.-M., Rosiau E., Crépeau M.-J., Surcouf O., Esquivel-Rodriguez J., Marcelo P., Mareck A., Guérineau F. (2015). Tuning of Pectin Methylesterification: Pectin methylesterase inhibitor 7 modulates the processive activity of co-expressed pectin methylesterase 3 in a pH-dependent manner. J. Biol. Chem..

[12] Hocq L., Sénéchal F., Lefebvre V., Lehner A., Domon J.-M., Mollet J.-C., Dehors J., Pageau K., Marcelo P., Guérineau F. (2017). Combined Experimental and Computational Approaches Reveal Distinct pH Dependence of Pectin Methylesterase Inhibitors. Plant Physiol..

[13] Rhee S.Y., Osborne E., Poindexter P.D., Somerville C.R. (2003). Microspore separation in the quartet 3 mutants of Arabidopsis is im-paired by a defect in a developmentally regulated polygalacturonase required for pollen mother cell wall degradation. Plant Physiol..

[14] Lionetti V., Cervone F., De Lorenzo G. (2015). A lower content of de-methylesterified homogalacturonan improves enzymatic cell separation and isolation of mesophyll protoplasts in Arabidopsis. Phytochemistry.

[15] Tieman D.M., Handa A.K. (1994). Reduction in Pectin Methylesterase Activity Modifies Tissue Integrity and Cation Levels in Ripen-ing Tomato (Lycopersicon esculentum Mill.) Fruits. Plant Physiol..

[16] Bouton S., Leboeuf E., Mouille G., Leydecker M.T., Talbotec J., Granier F. (2002). QUASIMODO1 encodes a putative mem-brane-bound glycosyltransferase required for normal pectin synthesis and cell adhesion in Arabidopsis. Plant Cell..

[17] Gao P., Xin Z., Zheng Z.L. (2008). The OSU1/QUA2/TSD2-encoded putative methyltransferase is a critical modulator of carbon and nitrogen nutrient balance response in Arabidopsis. PLoS ONE.

[18] Krupková E., Immerzeel P., Pauly M., Schmülling T. (2007). The TUMOROUS SHOOT DEVELOPMENT2 gene of Arabidopsis encod-ing a putative methylotransferase is required for cell adhesion and coordinated plant development. Plant J. Cell Mol. Biol..

[19] Mouille G., Ralet M.-C., Cavelier C., Eland C., Effroy D., Hématy K., McCartney L., Truong H.N., Gaudon V., Thibault J.-F. (2007). Homogalacturonan synthesis in Arabidopsis thaliana requires a Golgi-localized protein with a putative methyltransferase domain. Plant J..

[20] Neumetzler L., Humphrey T., Lumba S., Snyder S., Yeats T.H., Usadel B., Vasilevski A., Patel J., Rose J.K.C., Persson S. (2012). The FRIABLE1 Gene Product Affects Cell Adhesion in Arabidopsis. PLoS ONE.

[21] Du J., Kirui A., Huang S., Wang L., Barnes W.J., Kiemle S.N., Zheng Y., Rui Y., Ruan M., Qi S. (2020). Mutations in the Pectin Methyltransferase QUASIMODO2 Influence Cellulose Biosynthesis and Wall Integrity in Arabidopsis. Plant Cell.

[22] Cannon M.C., Terneus K., Hall Q., Tan L., Wang Y., Wegenhart B.L. (2008). Self-assembly of the plant cell wall requires an exten-sin scaffold. Proc. Natl. Acad. Sci. USA.

[23] Smallwood M., Martin H., Knox J.P. (1995). An epitope of rice threonine- and hydroxyproline-rich glycoprotein is common to cell wall and hydrophobic plasma-membrane glycoproteins. Planta.

[24] Hall H.C., Cheung J., Ellis B.E. (2017). Immunoprofiling reveal unique cell-specific pattern of wall epitopes in the expanding Ara-bidopsisstem. Plant J..

[25] Verger S., Chabout S., Gineau E., Mouille G. (2016). Cell adhesion in plants is under the control of putativeO-fucosyltransferases. Development.

[26] Aeschbacher R.A., Hauser M.T., Feldmann K.A., Benfey P.N. (1995). The SABRE gene is required for normal cell expansion in Ara-bidopsis. Genes Dev..

[27] Pietra S., Gustavsson A., Kiefer C., Kalmbach L., Hörstedt P., Ikeda Y., Stepanova A.N., Alonso J.M., Grebe M. (2013). Arabidopsis SABRE and CLASP interact to stabilize cell division plane orientation and planar polarity. Nat. Commun..

[28] Walles B., Steeves T.A., Sussex I.M. (1991). 1989 Patterns in plant development. Nord. J. Botany.

[29] Javorka P., Raxwal V.K., Najvarek J., Riha K. (2019). artMAP: A user-friendly tool for mapping ethyl methanesulfonate-induced mu-tations in Arabidopsis. Plant Direct..

[30] Showalter A.M., Basu D. (2016). Extensin and Arabinogalactan-Protein Biosynthesis: Glycosyltransferases, Research Challenges, and Biosensors. Front. Plant Sci..

[31] Jones M.A., Shen J.-J., Fu Y., Li H., Yang Z., Grierson C.S. (2002). The Arabidopsis Rop2 GTPase Is a Positive Regulator of Both Root Hair Initiation and Tip Growth. Plant Cell.

[32] Carol R.J., Takeda S., Linstead P., Durrant M.C., Kakesova H., Derbyshire P., Drea S., Zarsky V., Dolan L. (2005). A RhoGDP dissociation inhibitor spatially regulates growth in root hair cells. Nat. Cell Biol..

[33] Ambrose C.S., Allard J.F., Cytrynbaum E.N., Wasteneys G.O. (2011). A CLASP-modulated cell edge barrier mechanism drives cell-wide cortical microtubule organization in Arabidopsis. Nat. Commun..

[34] Zhu C., Ganguly A., Baskin T.I., McClosky D.D., Anderson C.T., Foster C., Meunier K.A., Okamoto R., Berg H., Dixit R. (2015). The Fragile Fiber1 Kinesin Contributes to Cortical Microtubule-Mediated Trafficking of Cell Wall Components. Plant Physiol..

[35] Qiu J.-L., Jilk R., Marks M.D., Szymanski D.B. (2002). The Arabidopsis SPIKE1 Gene Is Required for Normal Cell Shape Control and Tissue Development. Plant Cell.

[36] McFarlane H.E., Young R.E., Wasteneys G.O., Samuels A.L. (2008). Cortical microtubules mark the mucilage secretion domain of the plasma membrane in Arabidopsis seed coat cells. Planta.

[37] Redei G.P., Koncz C., Chua N.-H., Koncz C., Schell J. (1992). Classical mutagenesis. Methods in Arabidopsis Research.

[38] Kohorn B.D., Hoon D., Minkoff B.B., Sussman M.R., Kohorn S.L. (2016). Rapid Oligo-Galacturonide Induced Changes in Protein Phos-phorylation in Arabidopsis. Mol. Cell. Proteom..

[39] Thorvaldsdottir H., Robinson J.T., Mesirov J.P. (2013). Integrative Genomics Viewer (IGV): High-performance genomics data visuali-zation and exploration. Brief Bioinform..

[40] Blumenkrantz N., Asboe-Hansen G. (1973). New method for quantitative determination of uronic acids. Anal. Biochem..

[41] Krupkova E., Immerzeel P., Pauly M., Schmulling T. (2007). The TUMOROUS SHOOT DEVELOPMENT2 gene of Arabidopsis encod-ing a putative methyltransferase is required for cell adhesion and co-ordinated plant development. Plant J..

[42] Rozewicki J., Li S., Amada K.M., Standley D.M., Katoh K. (2019). MAFFT-DASH: Integrated protein sequence and structural alignment. Nucleic Acids Res..

[43] Bienert S., Waterhouse A., De Beer T.A.P., Tauriello G., Studer G., Bordoli L., Schwede T. (2017). The SWISS-MODEL Repository—new features and functionality. Nucleic Acids Res..

