# Supplementary material for: Pectin Dependent Cell Adhesion Restored by a Mutant Microtubule Organizing Membrane Protein
Source: Plants (Basel). 2021 Apr 2;10(4):690. doi: 10.3390/plants10040690 (PMC8067205; doi:10.3390/plants10040690)
Supplement: Supplementary file 1 [file plants-10-00690-s001.zip › supp rev/Kohorn et al Spp Figs.pdf]

Figure S1

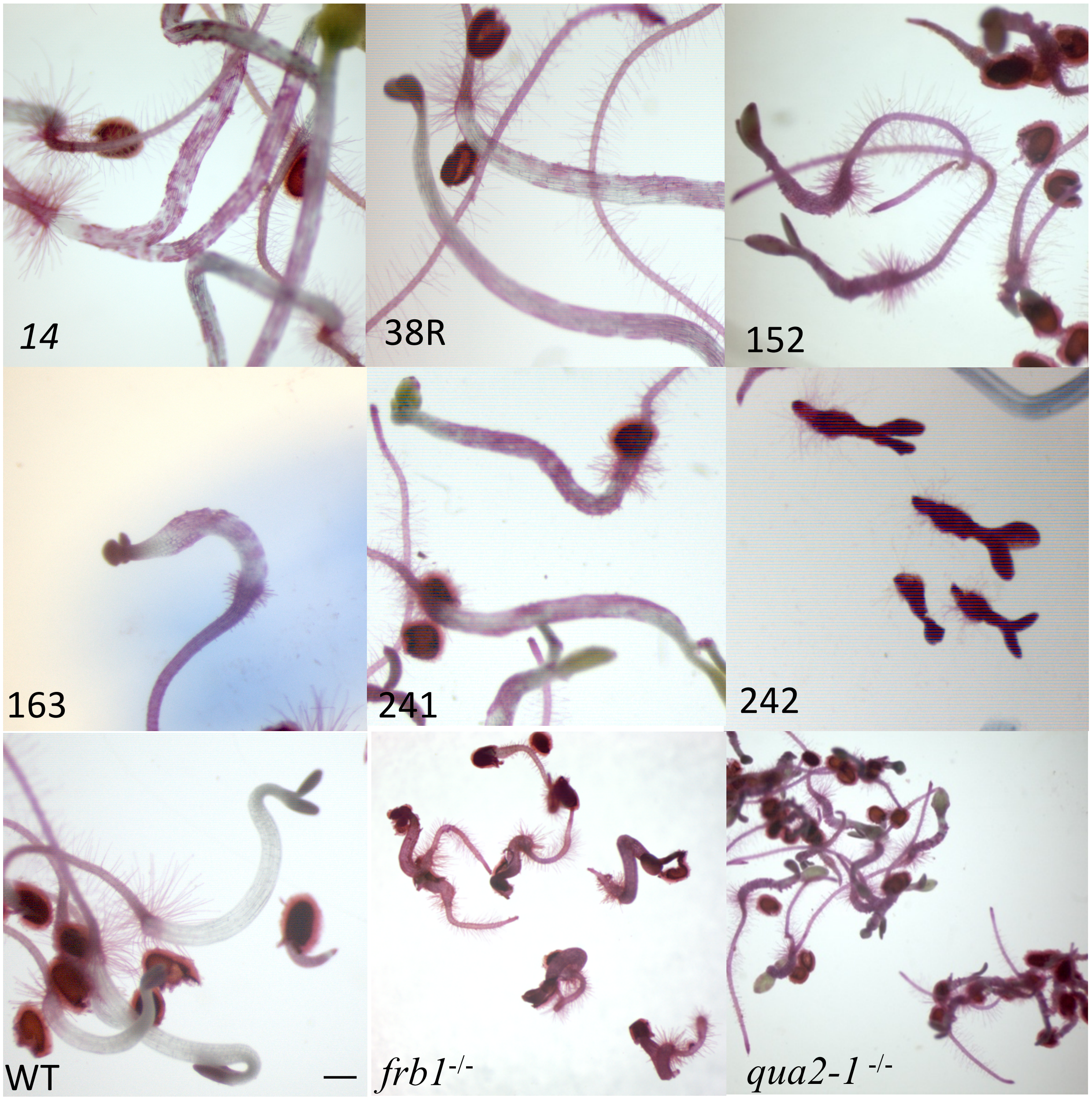

Figure S1. Ruthenium Red stained M2 dark grown hypocotyls from the indicated seed pool. Shown are red staining seedlings isolated from 192 M2 pools of seeds of 20-33-plants. WT, *frb1* and *qua2* are shown as controls. This report describes the characterization of mutant 38R, and 14, 152, 163 241 and 242 will be reported elsewhere. Bar indicate 0.5mm

Figure S2

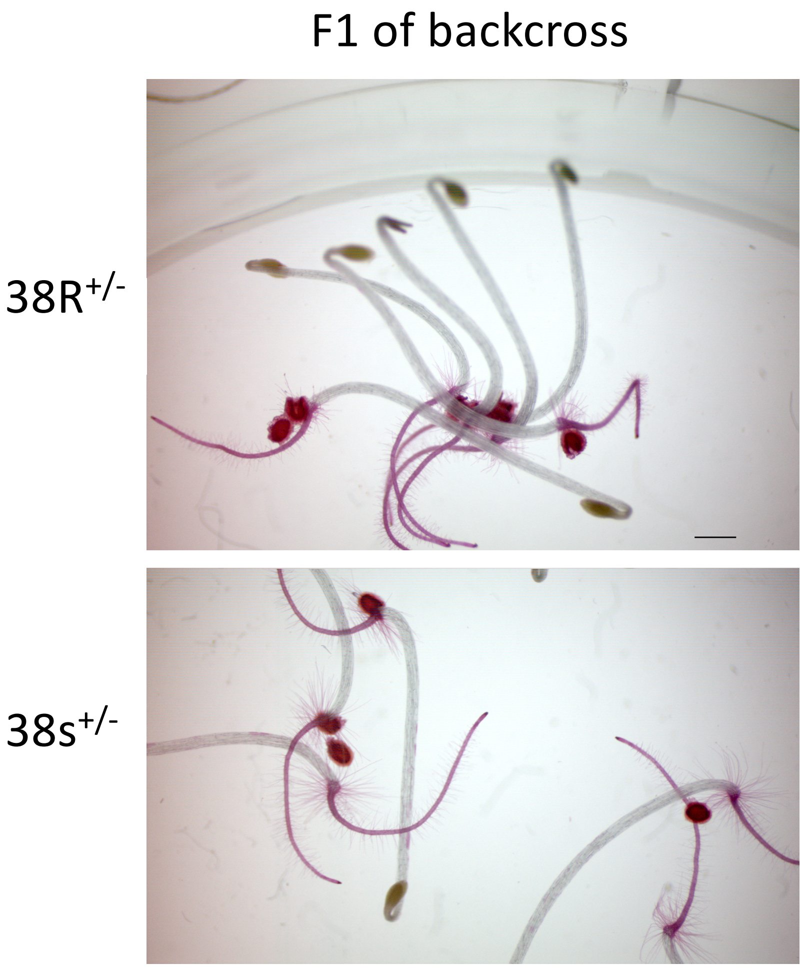

Figure S2. 38R and 38S are recessive alleles. Shown are Ruthenium Red stained dark grown hypocotyls of the F1 of the indicated mutant crossed to WT. Bar indicates 1 mm.

Figure S3

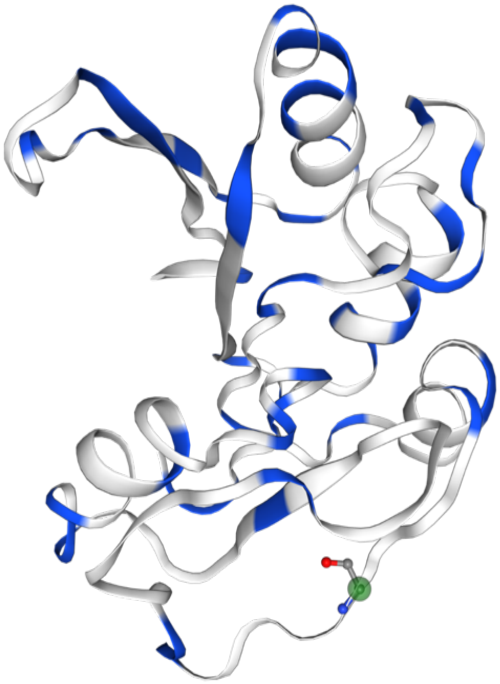

38R/qua2-4
(Gly580→Glu)
qua2-1
(Ile625 → STOP)

```
QUA2        _ MSMPLQRGISGVRVSDSSDDLRDSQMKDKTERARSTENNNLTLRFPFGFLFSNQSSSKHG 60
At1g13860   _ M------KVASV------------------------------------------------ 6
At2g03480   _ MRGSWYKSVSSV------------------------------------------------ 12
              *       ::.*

QUA2        _ GGGENGFSADPYSARSRHRLMLLFLKISLVLIVVIALAGSFWWTISISTSSRGHVYHNYR 120
At1g13860   _ -----------IGLRPRISGLLFLTLGVIALITILVPNSDSSSTTSTTRVPPSNIYSNYG
At2g03480   _ -----------FGLRPRIRGLLFFIVGVVALVTILAPLTSNSYDSSSSSTLVPNIYSNYR
                          . *.*   :*::    :.*:.::.   .     * :     ::* **

QUA2        _ RLQEQLVSDLWDIGEISLGPNRWKELEYCNIESENFVPCFNVSENLALGYSNGDENDRFC 180
At1g13860   _ RVKEQAAVDYLDLRFFSLGVNRLKEFPLCGKERDNYVPCYNVT-----------ESDRNC 104
At2g03480   _ RIKEQAAVDYLDLRSLSLGAS-LKEFPFCGKERESYVPCYNITGNLLAGLQEGEELDRHC 120
              *::** . *  *:  :*** .  **:  *. * :.:***:*::           * ** *

QUA2        _ GPGSKQE-CLELPPVKYRVPLRWPTGKDIIWHSNVKITAQEVVSSGSITKRMMMMEDDQI 239
At1g13860   _ EFAREEERCLVRPPRDYKIPLRWPVGRDIIWTGNVKITKDQFLSSGTMTKRLMLLEENQI 167
At2g03480   _ EFEREKERCVVRPPRDYKIPLRWPLGRDIIWSGNVKITKDQFLSSGTVTTRLMLLEENQI 180
                  ::* *:  ** .*::***** *:**** .***** ::.:***::*.*:*::*::**

QUA2        _ SFRSASPMS-DEVEDYSHQIAEMIGIKKDN-FIEAGVRTILDIGCGYGSFGAHLLSKQIL 297
At1g13860   _ TFHSDDGLIFDGVKDYAFQIAEMIGLGSDTEFPQAGIRTVLDIGCGFGSFGAHLVSLNVM 224
At2g03480   _ TFHSEDGLVFDGVKDYARQIAEMIGLGSDTEFAQAGVRTVLDIGCGFGSFGAHLVSLKLM 240
              :*:* . :  * *:**: *******: .*. * :**:**:******:*******:* :::

QUA2        _ TMCIANYEASGSQVQLTLERGLPAMIGSFISKQLPYPSLSFDMLHCLRCGIDWDQKDGLL 357
At1g13860   _ PICIAEYETSGSQVQLALERGLPAMIGNFFSKQLPYPALSFDMVHCAQCGITWDIKDAML 284
At2g03480   _ PICIAEYEATGSQVQLALERGLPAMIGNFFSKQLPYPALSFDMVHCAQCGTTWDIKDAML 300
              .:***:**::******:**********.*:*******:*****:** :**  ** **.:*

QUA2        _ LVEIDRVLKPGGYFVWTSPLTNPR-NKDHLKRWNF---VHDFAESICWTLLNQQDETVVW 413
At1g13860   _ LLEVDRVLKPGGYFVLTSPTSKAQGNSPDTKKTSISTRVDELSKKICWSLSGQQDETFLW 344
At2g03480   _ LLEVDRVLKPGGYFVLTSPTNKAQGNLPDTKKTSISTRVNELSKKICWSLTAQQDETFLW 360
              *:*:*********** *** .:.: *  . *: .:   *.::::.***:*  *****.:*

QUA2        _ KKTINTKCYSSRKPGVGPSVCTKGHDVESPYYRPLQMCIGGTRSRRWIPIEGRTRWPSRS 473
At1g13860   _ QKTADPNCYSSRSQASIP-VCKDDDSV--PYYHPLVPCISGTKSKRWIPIQNRSR---AS 398
At2g03480   _ QKTSDSSCYSSRSQASIP-LCKDGDSV--PYYHPLVPCISGTTSKRWISIQNRSA---VA 414
              :** :..*****. .  * :*.....*  ***:**  **.** *:***.*:.*:     :

QUA2        _ NMNKTELSLYGLHPEVLGEDAENWKITVREYWSLLSPLIFSDHPKRPGDEDPSPPYNMLR 533
At1g13860   _ GTSLSELEIHGIKPEEFDEDIQVWRSALKNYWSLLTPLIFSDHPKRPGDEDPVPPFYMIR 456
At2g03480   _ GTTSAGLEIHG-------------KSALKNYWSLLTPLIFSDHPKRPGDEDPLPPFNMIR 459
              .  . : *.::*             : ::::*****:**************** **: *:*

QUA2        _ NVLDMNAQFGGLNSALLEARKSVWVMNVVPTAGPNHLPMILDRGFVGVLHNWCEPFPTYP 593
At1g13860   _ NAMDMNARYGNLNQALLNQGKSVWVMNVVPVKARNTLPIILDRGFTGALHDWCEPFPTYP 518
At2g03480   _ NVMDMHARFGNLNAALLDEGKSAWVMNVVPVNARNTLPIILDRGFAGVLHDWCEPFPTYP 521
              *.:**:*::*.** ***:  **.*******. . * **:******.*.**:*********

QUA2        _ RTYDLVHADNLLSLQTSQPRKTCLLIDIFTEIDRLLRPEGWVIIRDTAQLVEKARETITQ 653
At1g13860   _ RTYDMLHANELLTHLSSE---RCSLMDLFLEMDRILRPEGWVVLSDKLGVIEMARTLAAR 575
At2g03480   _ RTYDMLHANELLTHLSSE---RCSLMDLFLEMDRILRPEGWVVLSDKVGVIEMARALAAR 578
              ****::**::**:  :*:    * *:*:* *:**:*******:: *.  ::* **   ::

QUA2        _ LKWEARVIEVESSSEQRLLICQKPFTKRQSI 684
At1g13860   _ VRWEARVIDIQDGSDQRLLVCQKPLLKK--- 603
At2g03480   _ VRWEARVIDLQDGSDQRLLVCQKPFIKK--- 606
              ::******:::..*:****:****: *:
```

Figure S3. CLUSTAL format alignment of QUA2 amino acid sequence to its two closest *Arabidopsis thaliana* methyl transferase homologous (*9*). An asterisk indicates positions which have a single, fully conserved residue, a colon indicates conservation between groups of strongly similar properties, and a period indicates conservation between groups of weakly similar properties (*10*). The location of *qua2-4* (G to E) is highlighted in green and *qua2-1* (I to STOP) is highlighted in red. Swiss-Model predicted structure of aa 532-678, which lie within the methyl transferase domain of QUA2, is shown to the left of the sequence. Hydrophobic regions are highlighted in white, while hydrophilic regions are highlighted in blue (*11*). *qua2-4* (green dot at amino acid 580) lies within a long hydrophobic region of QUA2 and is characterized by a change from a glycine (aa structure shown) to a glutamic acid.

Figure S4

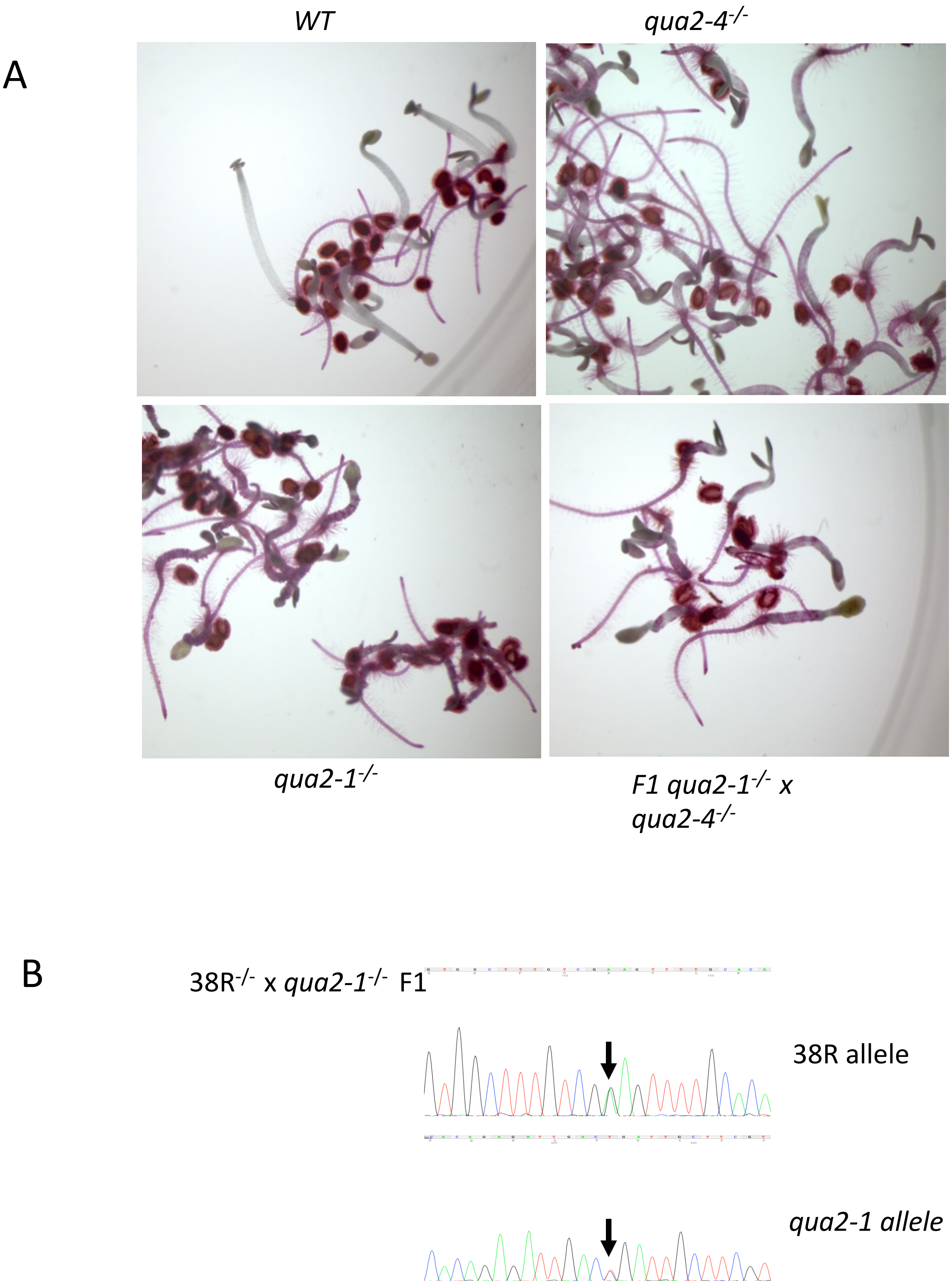

Figure S4. *qua2-1* does not complement 38R adhesion defect. **A**) 4 day old Ruthenium Red stained dark grown hypocotyls from the indicated genotype. B) The *QUA2* gene was PCR amplified and sequenced from DNA of an F1 *qua2-1*$^{-/+}$ *qua2-4*$^{-/+}$ seedling, and shown are the heterozygous loci for each allele.

Figure S5

38S $^{+/-}$ x *sab1-5* $^{+/-}$ F1

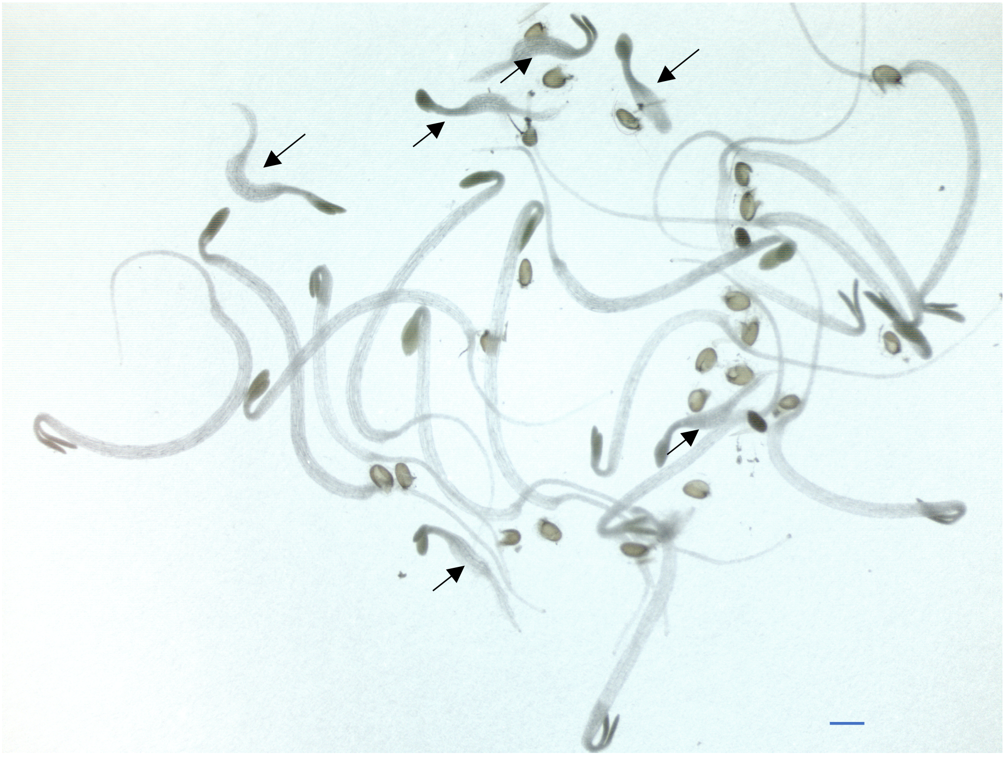

Figure S5. 38S and *sab1-5* are alleles of the same gene. Shown are dark grown hypocotyls from F1 seeds of a 38S$^{-/+}$ x sab1-5$^{-/+}$ cross. The presence of 1/7th of the population of stunted progeny (arrows) indicates the lack of complementation. Bar indicates 1 mm.

Figure S6 . Excel file of RNA seq
List of differentially expressed genes related to the cell wall compared between *sab* and WT. Similar levels are seen in *sab/qua* vs WT. In addition the following genes were evaluated and found not to differ significantly between genotype; Cell Wall Integrity- *FER1, HERK1, HERK2, FEI1, FEI2,KIN13A, THE1, WAK1-5*. Biosynthesis; *CESA, GAE1-6, PMT, PG, PGIP, PGX1, MNA, ORT2, APG*.

Figure S7 (compressed) Additional Excel sheets 2-4 show Differential Expressed Genes (DEG) between WT and *sab*, *qua2* or *sab qua2*.
